# Botulinum Toxin and Neuronal Regeneration after Traumatic Injury of Central and Peripheral Nervous System

**DOI:** 10.3390/toxins12070434

**Published:** 2020-07-02

**Authors:** Siro Luvisetto

**Affiliations:** Institute of Biochemistry and Cell Biology, National Research Council of Italy, via Ramarini 32, Monterotondo Scalo, 00015 Rome, Italy; siro.luvisetto@cnr.it

**Keywords:** botulinum neurotoxin, peripheral nervous system, central nervous system, peripheral nerve injury, spinal cord injury, nerve regeneration

## Abstract

Botulinum neurotoxins (BoNTs) are toxins produced by the bacteria *Clostridium* *botulinum*, the causing agent for botulism, in different serotypes, seven of which (A–G) are well characterized, while others, such as H or FA, are still debated. BoNTs exert their action by blocking SNARE (soluble N-ethylmale-imide-sensitive factor-attachment protein receptors) complex formation and vesicle release from the neuronal terminal through the specific cleavage of SNARE proteins. The action of BoNTs at the neuromuscular junction has been extensively investigated and knowledge gained in this field has set the foundation for the use of these toxins in a variety of human pathologies characterized by excessive muscle contractions. In parallel, BoNTs became a cosmetic drug due to its power to ward off facial wrinkles following the activity of the mimic muscles. Successively, BoNTs became therapeutic agents that have proven to be successful in the treatment of different neurological disorders, with new indications emerging or being approved each year. In particular, BoNT/A became the treatment of excellence not only for muscle hyperactivity conditions, such as dystonia and spasticity, but also to reduce pain in a series of painful states, such as neuropathic pain, lumbar and myofascial pain, and to treat various dysfunctions of the urinary bladder. This review summarizes recent experimental findings on the potential efficacy of BoNTs in favoring nerve regeneration after traumatic injury in the peripheral nervous system, such as the injury of peripheral nerves, like sciatic nerve, and in the central nervous system, such as spinal cord injury.

## 1. Introduction

Regeneration and recovery of nerve tissues are a great challenge for medicine, because they heavily influence patients’ quality of life. During embryogenesis, neurons have the ability to proliferate [1]; at the beginning of postnatal life most neurons quickly and definitively lose this ability and the nervous tissue becomes unable to regenerate after neuronal lesions [2,3]. Neuronal lesions are very common and can result from a variety of pathological and non-pathological states, including neurodegenerative diseases, such as amyotrophic lateral sclerosis, Parkinson’s disease, Alzheimer’s disease, Huntington’s disease, and diabetic neuropathy causing progressive loss of neuronal structure and/or neuronal function. Neuronal lesions may also be the consequence of vascular diseases; one of the most common is stroke, where a substantial reduction in blood flow to some parts of the brain causes cerebral death of brain tissue [4]. Impairment of brain function may result in transient or lasting uni- or bilateral paralysis on one or both sides of the body, difficulties in speaking or eating, and loss of muscular coordination.

Relevant causes of neuronal lesions also include direct injuries to the nervous system due to physical traumas. Traumatic damages to the central nervous system (CNS), both at the brain and spinal cord, often cause irreversible effects and current treatment strategies do not offer safe results [5,6,7]. Unlike neurons of the CNS, the nerve fibers of the peripheral nervous system (PNS) have the ability to grow back after traumatic injury [8,9] with high reparative and regenerative activity [10,11]. 

Due to the vastness of this topic, only the neuronal damages due to direct traumatic injuries on the nervous system will be considered in this review. In particular, greater attention will be focused on therapeutic approaches, both current and promising for the future, using botulinum neurotoxins (BoNTs), useful to treat the consequences of these traumatic injuries. In the first part of this review (Section 2 and related subsections), experimental and clinical data will be presented, with particular emphasis on the beneficial effects of botulinum neurotoxins in promoting axonal regeneration after traumatic peripheral nerve injury (PNI). In the second part of this review (Section 3 and related subsections) some promising outcomes observed after treatment with botulinum neurotoxins in animal models of spinal cord injury (SCI) will be discussed.

## 2. Peripheral Nervous System

The PNS refers to the part of the nervous system outside the brain and spinal cord. The primary role of the PNS is to connect the CNS to the organs, limbs, and skin. The PNS includes cranial (12 pairs) and spinal (31 pairs) nerves, their roots and branches, and the neuromuscular junctions. The PNS allows the brain and spinal cord to receive and send information to other areas of the body allowing responses to stimuli of the environment. The PNS itself is divided into two parts: the somatic nervous system (i.e., the part of PNS responsible for carrying sensory and motor information to and from the CNS) and the autonomic nervous system (i.e., the part of the PNS responsible for regulating involuntary body functions, such as blood flow, heart rate, digestion, and breathing). The autonomic nervous system is further divided into three separate parts: the parasympathetic, sympathetic, and enteric nervous system.

In the PNS, each neuron has a long process, known as the axon, which runs together in bundles called fibers, and multiple fibers form a nerve. Nerves often extend a great length from the central nervous system to reach the periphery of the body. For example, the longest nerve in the human body, the sciatic nerve, originates around the lumbar region of the spine and its branches reach until the tip of the toes, measuring a meter or more in an average adult. The nerves also contain connective tissue and blood vessels and are classified based on the types of neurons they contain—sensory, motor or mixed nerves (if they contain both sensory and motor neurons)—as well as the direction of information: afferent nerves contain neurons that bring information to the central nervous system, while efferent nerves contain neurons that transmit the signals originating from the central nervous system to the organs and muscles, and put into action the orders from the brain.

Injuries can occur at any point in peripheral nerves resulting in a loss of function of the parts of the body that the nerves reach. Thus, it is of fundamental importance to understand how the nerves, or even how the axonal structure within the nerves, are protected from the mechanical stresses exerted on them and how they respond to insult and restore original conditions before the occurrence of the damage or, in other words, how they regenerate.

### 2.1. Outline of Peripheral Nerve Anatomy

**Figure 1:**
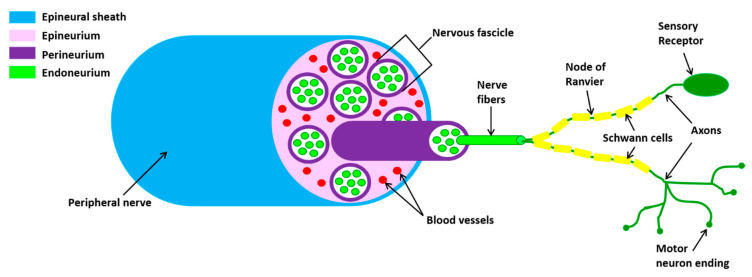
Schematic representation of the peripheral nerve structure. Axons, surrounded by myelinating Schwann cell sheaths, are enclosed by endoneurium. The perineurium binds individual axons together to form fascicles. Several axons are contained in each fascicle. The epineurium groups fascicles to one another, forming the nerve cable.

Peripheral nerves are composed of sensory and motor neurons whose long axons communicate with distant target organs. The cell bodies of sensory neurons are located in the dorsal root ganglion while those of the motor neurons are found within the CNS, the spinal cord or brainstem. The axons of the peripheral nerve are wrapped by a complex coating system consisting of three distinct layers (Figure 1).

The peripheral nerves are enveloped by connective tissue (the epineurium) with a large circular outer layer and septa penetrating between the fascicle. The latter are wrapped by one or more layers of endothelial-like cells (the perineurium). The fascicles contain dozens or hundreds of myelinated and non-myelinated axons. The myelinated axons have three sheaths produced by Schwann cells: (1) the myelin sheath, an insulating structure consisting of numerous layers of the cell membrane; (2) the Schwann cell (SC) sheath, consisting of the cytoplasm of the cell which wraps the axon and the myelin sheath; (3) the endoneural tube (the endoneurium), an uninterrupted tube that accompanies the neuritis from its origin to the terminal organs. The endoneurium is continuous while the myelin and SC sheaths are interrupted in the Ranvier nodes. A fine network of capillaries exists in association with the endoneurium.

### 2.2. Pathophysiology of Traumatic Injuries of Peripheral Nerve

Trauma of peripheral nerve can be of different origins. Mechanical processes, such as nerve compression, stretching due to intense physical activity or fractures, sharp injuries due to cutting objects, and so on, can lead to lesions with different degrees of severity, ranging from the blockage of nerve conductivity, focal demyelination of the axons, ischemic phenomena, increased neuropeptide production, increased spinal dorsal horn circuits activity involved in pain perception, to a complete loss of continuity with nerve lacerations or complete transections. It should be pointed out that traumatic damage of nerves is often accompanied by vascular damage that can lead to ischemia, occlusion of arteries from which the vasa nervorum is derived or hemorrhage occurring within the nerve sheaths. This vascular dysfunction and consequent hypoxia contribute to the manifestation of neuropathic pain. 

Success in regeneration depends also on the extent of peripheral nerve injury. Grading systems were developed in order to correlate the microscopic changes of the injured nerve with the clinical manifestations and prognosis. Seddon [12] first classified nerve injuries into three categories, named neuropraxia, axonotmesis, and neurotmesis, respectively, ordered from the least to the most severe injury. Neuropraxia consists of a temporary block of conduction without loss of nerve continuity: axons are anatomically intact, but nonfunctional. Usually, no signs of Wallerian degeneration [13] are identified and neuropraxia allows for rapid and spontaneous healing. Axonotmesis is a more severe damage with Wallerian degeneration: axon and surrounding myelin sheath are completely disrupted while perineurium and epineurium remain intact. Despite this, since the endoneural tube is not interrupted, axons can regenerate inside it spontaneously and the prognosis of recovery is excellent. Neurotmesis results in a total disconnection between the two ends of the injured nerve: the endoneural tube and all the nerves are interrupted so that spontaneous regeneration is not possible and surgery is needed. The functional loss is complete and recovery without surgical intervention, or any other alternative, is unlikely due to the intense scarring phenomena and loss of the connective coatings functioning as a guide to axonal regrowth.

Sunderland [14] expanded the classification of Seddon [12] to five grades to distinguish the extent of damage in connective tissues. Under this classification, neuropraxia (grade 1) and neurotmesis (grade 5) were maintained while axonotmesis was further divided into three grades. First grade of axonotmesis (grade 2 of Sunderland’s classification) is related to damage in which the axon and myelin sheath become disconnected but connective tissues’ continuity remain conserved. Second grade of axonotmesis (grade 3 of Sunderland’s classification) indicates a damage where the axon and axonal sheath become disconnected along with the endoneurial layer, whereas the layers of connective tissues remain intact. In the third grade of axonotmesis (grade 4 of Sunderland’s classification), only a continuity of epineurium is maintained whereas all the other layers and axonal sheath become disconnected. Finally, Mackinnon and Dellon [15] introduced a sixth degree of injury, which is a combination of the five degrees of injury of Sunderland’s classification [14]. 

### 2.3. Mechanisms of Nerve Regeneration after Peripheral Injuries

Mechanisms of regeneration of injured peripheral nerves is nowadays much better known due to the continuously expanding knowledge on neurogenesis, molecular biology, physiology of PNS, and pathophysiology of healing [16]. Following a lesion, the axonal soma can regenerate the peripheral stump thanks to the axoplasmic interaction with SCs that wrap the nerve fibers. Since SCs maintain the capacity to divide and enter in a reproductive cycle, they are recognized as one of the main actors responsible for regeneration in the PNS. During Wallerian degeneration SCs divide and begin to form a cell “tube” that guides the regeneration while macrophages engulf debris. Moreover, each nerve is in contact with a small number of satellite cells that are in contact with the capillaries that have a nutritional function. Cytosolic proteins and neurotransmitters are conveyed on the periphery from the axonal flow by means of driving molecules (dynein and kinesin) that drag the membranous vesicles containing proteins along microtubules. Axonal flow also brings growth factors coming from the terminal organs and the return of unused substances from synapses and enzymes from the site of the injury. 

Altogether, these processes take some time to reform the connection and, normally, the restoring of functionality may take months. Nevertheless, poor functional recovery may occur due to chronic SC denervation, chronic neuronal axotomy, and misdirection of regenerating axons into wrong endoneurial tubes. Moreover, due to lack of physical activity, muscle atrophies. Finally, peripheral nerve damage removes a source of sensory input from the somatosensory system that leads to long-term changes in spinal somatosensory functions. As a consequence, neuropathic pain develops in the partially denervated region.

The spontaneous nerve repair may not be sufficient to achieve proper functional recovery [17], and regeneration of PNI is aided by many different types of treatment [18,19]. The primary medical therapy for complete lesions is an end-to-end repair by suturing of nerve stumps via epineurial and/or group fascicular suturing. In the case of a significant nerve gap formation where end-to-end repair is not possible, peripheral nerve grafts (autografts, allografts or xenografts) and nerve conduits (synthetic, biological or hybrid) and tubulization are required to serve as a bridge between the nerve stumps across the gap and to support axonal regrowth [20,21,22,23,24,25]. Other approaches, ranging from cell-based therapy [26], electrical stimulation [27] or pharmacological medications [19], are also used. In particular, pharmacological medications, which essentially include analgesics, corticosteroids, and opioids, are helpful to relieve pain but, unfortunately, they are not useful to treat PNIs because they cannot accelerate the nerve regeneration/functional recovery. Last but not least, unexpected results have been obtained by functional reeducation through physiotherapy and physical activity [28,29,30]. It should be noted that physiotherapy forms an essential part of the treatment used for initiation and support of peripheral nerve regeneration.

### 2.4. Botulinum Toxin and Nerve Regeneration after Peripheral Injuries

BoNTs are bacterial toxins produced by anaerobic bacteria of clostridium genus in seven different serotypes, termed BoNT/A to BoNT/G, and many variants [31]. Additional BoNT serotypes are occasionally found [32,33,34,35], but the recognition of them as new serotypes is still a matter of debate awaiting confirmation. All seven serotypes share a similar mechanism of action, based on BoNT-induced blockade of neurotransmitters release after specific binding and entry into the cytosol of nerve terminals. Neurotransmitters release is prevented by enzymatic cleavage of different protein components of the SNARE complex, depending on the serotype, involved in presynaptic neurotransmitter release. Further description of the mechanism of binding, internalization, and mode of action of the different BoNTs serotypes is beyond the purpose of this review and the reader is strongly encouraged to refer to some excellent reviews present in the literature. For example, see the reviews of Rossetto et al. [36], Pirazzini et al. [37] or Rasetti-Escargueil et al. [38], which cover all the biological, pharmacological, and toxicological aspects related to the use of BoNTs.

The medical use of BoNTs, as treatment for well-defined pathological conditions, derived from studies on the pathophysiology of botulism and, in particular, on the mechanism of action of BoNT/A. In fact, the observation that injection of BoNT/A in muscle selectively inhibits the release of acetylcholine (ACh) evoked by cholinergic nerve endings at the neuromuscular junction, has paved the way for the use of BoNT/A as a therapeutic agent in a variety of neurological disorders caused by hyperfunctionality of cholinergic terminals, such as dystonia, spasticity, and muscle spasms [39]. In parallel, thanks to its activity on the muscles, BoNT/A has become a “cosmetic” drug to remove facial wrinkles, due to the activity on the mimic muscles, and its use exponentially expanded as an extraordinary “aesthetic miracle”. Finally, after the observation that many patients, treated for cervical dystonia, referenced the analgesic effect on headaches, many efforts have been made to study the potential therapeutic action of BoNT/A against pain [40,41,42,43,44,45]. Nowadays, BoNT/A is recognized as effective at reducing pain in a number of disease states, including migraines, some form of neuropathic pain, lower back pain, myofascial pain, and bladder pain [46,47,48,49,50]. A similar fate happened to BoNT/B, with the difference that for this toxin the therapeutic use is limited to fewer pathologies than BoNT/A.

It should be remembered here that many neurophysiological changes occur after intramuscular injection of toxin, as recently described in detail by Palomar and Mir [51]. Clinical studies presented in this review demonstrate the indirect action of the toxin on the excitability of spinal cord circuitry in humans, by acting on the pre-synaptic neuromuscular junction and by decreasing spindle afferent input to the spinal cord. The results also show that BoNT injected at therapeutic doses induces spinal and cortical effects through plastic rearrangement in the brain subsequent to denervation or alterations in sensory input. An excellent clinical example is the positron emission tomography (PET) study of the pattern of cortical activation induced by BoNT injections. PET activation studies show an over-activity of striatum and non-primary motor areas with under-activity of the primary motor cortex of dystonia patients during voluntary movements. BoNT injections in those patients improved writing technique together with increased activation in the parietal cortex and caudal supplementary motor area, leaving the pattern of activity in the primary motor cortex unchanged. These results clearly show a cortical reorganization secondary to the BoNT-induced changes in motor strategy.

Experimental evidences in favor of regenerative effects of BoNTs on injured peripheral nerves came from studies on animal models [40], which are useful to investigate the mechanisms related to axonal regeneration and tissue reinnervation [52]. Animal model studies have been mainly performed with 150 kDa purified BoNT/A and BoNT/B, or equivalent commercial products. In the following, BoNT/A and BoNT/B will indifferently indicate the serotype of toxin used regardless of which preparation was actually used.

A common consequence of a peripheral nerve injury is the onset of neuropathic pain, a chronic pain resulting from damage or diseases of somatosensory nervous system [53]. Examples of neuropathic pain in humans include postherpetic neuralgia, diabetic neuropathy, complex regional syndrome, pain associated with spinal cord injury, and many other pathological conditions. In a case report article, Klein [54] was the first to describe the ability of BoNT/A to alleviate the symptoms of neuropathic pain. Starting from this observation, basic science studies began with the aim to define the analgesic role of BoNTs and the mechanisms involved. Historically, the effect of BoNT/A on neuropathic pain was first analyzed in peripheral neuropathy induced by a partial sciatic nerve transection in rats [55], followed by a study on the effect of BoNT/A on neuropathic pain induced by ligation of the spinal nerve L5/L6 [56] and, finally, by a study of BoNT/A on neuropathic pain induced by chronic constriction (CCI) of the sciatic nerve [57]. In all these studies, a single peripheral BoNT/A injection, into the hind paw plantar surface, was sufficient to reduce the mechanical and/or thermal hyperalgesia of the injured hind paw, and this effect lasted for at least two weeks after the injection. It should be remembered that mechanical and thermal hyperalgesia are distinctive properties of experimental models of peripheral neuropathies, being the classic behavioral manifestation of neuropathic pain in animals [58]. 

For completeness it must be remembered that Luvisetto et al. [57] and Marinelli et al. [59] injected a relatively high concentration of toxin, namely 15 pg/paw in CD1 mice weighing 35–40 g, compared to doses that induce analgesic effects in mice in other pain models [40], or as measured in other reports investigating BoNT/A toxicity [60,61]. However, in the cited reports [57,59], the authors reported no side effects in their experiments and the concentration used was considered safe. The discrepancy in the safety concentration of BoNT/A may essentially be dependent on the different toxin preparation which, as already mentioned, was not the commercial product but the 150 kDa toxin, self-prepared by the laboratory of Prof. Montecucco (University of Padua, Italy). The difference in BoNT/A toxicity can originate from the different batches of preparation.

Marinelli et al. [59] observed that BoNT/A not only reduced mechanical and thermal hyperalgesia in injured hind paw of mice and rats subjected to CCI, but also improved the functional recovery of the injured paw. The recovery was estimated by the increase of static sciatic index, a functional index derived from analysis of footprint walking tracks [62], and by the normalization of static weight-bearing, a functional index allowing an estimation of the percentage distribution of body weight between the ipsilateral hind paw and contralateral hind paw [63,64].

In addition to functional recovery, other data suggest the ability of BoNT/A to positively interfere with regenerative processes after peripheral nerve injury. The composite phenomena occurring after injury of peripheral nerves were described in previous paragraphs. Here it is useful to recall that after peripheral nerve injury, together with axonal degeneration, an infiltration of immune cells (macrophages and glial cells) occurs [65], with SCs having an important role [66]. The interaction of SCs with macrophages is essential to create a favorable environment for sprouting, elongation, and maturation of axons [67]. Moreover, nerve regeneration is associated with marked changes in gene and protein expression in sensory neurons. Accordingly, in sciatic nerve samples from mice subjected to CCI and treated with BoNT/A, Marinelli et al. [59] observed a significantly higher expression of Cdc2, a cyclin-dependent kinase that regulates the mitotic phase of the cell cycle [68] and the cell migration processes [69]. On the same line of evidence, Han et al. [70] demonstrated that after sciatic nerve injury, the SCs expressed an elevated Cdc2 level, together with an enhanced capacity to migrate, and that inhibition of Cdc2 blocked cell migration. The role of SCs in injured nerves is also favored by other peculiar processes. It is known that after nerve injury, the SCs dedifferentiate to immature SCs and enter into a proliferative state, acquiring again the expression of molecules typical of embryonic development [71], and gradually enhance the expression of S100β and glial fibrillary acidic proteins (GFAPs), proteins expressed by myelinating and non-myelinating SCs, respectively [72,73,74]. By immunofluorescence and Western blotting in the sciatic nerve, Marinelli et al. [59] showed an increased expression of regeneration associated proteins, such as Cdc2 and growth associated protein 43 (GAP-43), together with an enhanced expression SC proteins, such as S100β and GFAP, after BoNT/A treatment. 

These data suggest a direct interaction of BoNT/A with the SCs. The existence of an interaction of BoNT/A on regenerative processes throughout a direct action on the SCs was demonstrated by in vitro studies [75]. In such reports, the authors were able to ascertain that BoNT/A interacts with the proliferative state of SCs. In fact, as suggested by a historical study [76] and confirmed by Marinelli et al. [75], cultured SCs release ACh and proliferation of SCs strongly depends on the concentration of ACh in the cell culture medium, in the sense that a high concentration of ACh represses the proliferation of SCs [77]. The inhibition of the ACh-induced cell proliferation may depend on negative modulation of the neuregulin 1 (NRG1) levels. In fact, SC proliferation is mainly dependent on levels of NRG1: high levels of NRG1 usually increase SC proliferation [78]. Under this view, BoNT/A inhibits the ACh release from SCs because it acts as an “unlocking agent” on the blockage Ach induced of SC proliferation. Alternatively, it cannot be excluded that ACh released by injured nerves exerts a sort of bidirectional effect of ACh on SCs. In this context, it is possible that the BoNT/A blocks the release of ACh from the nerve, therefore the inhibitory effect of ACh on the proliferation of SC is however reduced. Further studies are necessary to better discriminate between the two possibilities.

The inhibition of ACh release from SCs by BoNT/A is compatible with the possibility that BoNT/A may induce its effects by cleaving SNAP-25 (synaptosomal-associated protein of 25 kDa) also in SCs. It should be reminded here that SNAP-25 protein is the protein of the SNARE complex selectively cleaved by BoNT/A. Evidences for the presence of SNAP-25 in SCs have been reported by Barden et al. [79], who found that in sympathetic varicosities in mouse vas deferens, the protein SNAP-25 together with other SNARE proteins is clustered with P2X receptors subunits and these receptor clusters are located in SCs. The possibility of a direct effect of BoNT/A on SCs supports the proposal that BoNT/A could be retrogradely transported from the site of injection along the nerve to reach the injured zone where it exerts its ability to influence regenerative processes by interacting directly with SCs. Demonstration of retrograde transport of BoNT/A from periphery to the CNS has been the object of intense research in recent years in many animal models (for review see [80]) and it has also been confirmed in CCI model [75,81]. 

In subsequent research, Cobianchi et al. [82] demonstrated that, after the complete crushing of the sciatic nerve, a single intraneural injection of BoNT/A increased the speed of axonal elongation and the number of regenerated myelin fibers. It should be noted that this regenerative effect is different from the motoneuronal sprouting reaction of motoneuronal terminal at the neuromuscular junction, due to a simulated denervation of intoxicated muscles [83]. However, histological evaluation showed a higher number and density of myelinated fibers in BoNT/A-injected nerves, suggesting an enhanced sprouting of regenerating myelinated fibers on the site of the nerve crush. On the other hand, BoNT/A did not affect the regeneration rate of unmyelinated axons, nor the collateral reinnervation of the paw skin by the intact neighboring saphenous nerve [82]. This study demonstrates another important feature of BoNT/A: in accordance with Lu and colleagues [84], the direct intraneural injection of the toxin causes no damage to the nerve, a difference from many other substances, such as local anesthetics [85]. Perineurial injection of BoNT/A is not limited to animal models and is under investigation in humans as well [86].

Similar to Cobianchi et al. [82], Irintchev et al. [87] observed that BoNT/A application to the axotomized femoral nerve modulates spinal responses and enhances motor recovery in rats. The authors applied BoNT/A only once using the time frame between axonal membrane damage and sealing, considering that this temporal window would be sufficient to “prime” the initial responses of motoneurons to injury, in particular their deafferentation, and, thus, eventually achieve long-term effects on regeneration without the need of repeated drug delivery to the injury site. As estimated by gait analysis, functional regeneration was enhanced already at two weeks after injury and recovery remained accelerated for months thereafter. The authors proposed that the improvement of regeneration in their model was a consequence of attenuated cholinergic input to femoral motoneurons with a possible neuroprotective effect of BoNT/A. It was also hypothesized that BoNT/A-related modulations of pain-related transmission may have positive functional consequences. 

With a different point of view, a recent study by Franz et al. [88] showed another interesting feature of the toxin in repairing the peripheral injuries: BoNT/A was able to reproduce the conditioning lesion effect, an effect characterized by an improvement of peripheral axon regeneration when the nerve lesion under consideration has been preceded by another nerve injury [89]. In detail, one week of BoNT/A conditioning treatment enhanced outgrowth of both murine motor axons in vivo, in a mouse tibial nerve injury model, and of human motor neuron neurites in vitro, in a human embryonic stem cell-based model. These findings have important implications in the clinic because BoNT/A preconditioning may be a treatment to enhance motor axon regeneration in nerve transfer surgery, a clinical condition in which healthy donor nerves are surgically redirected to restore function after nerve damage, such as from peripheral nerve injuries, plexopathies, and spinal nerve root avulsions [90]. However, in this context, ambiguous results have been obtained [91]. Authors found that motoneurons that are sprouting as a result of intramuscular injections of BoNT/A, into the tibialis anterior and extensor digitorum longus muscles, die after peripheral denervation of the affected muscles, favoring the view that motoneurons that are sprouting are vulnerable when challenged to grow further by a second stimulus. 

Although much research on the effects of BoNT/A in peripheral nerve regeneration is present in the literature, there is little or nothing for BoNT/B or other serotypes. To make up for this shortcoming, Finocchiaro et al. [92] performed a study where they compared the effects of BoNT/A and BoNT/B on the nerve regeneration processes in the CCI model of neuropathy. The authors found that BoNT/B, similarly to BoNT/A, can reduce neuropathic pain over a long period of time. However, the analgesic effects of BoNT/B, resulting from the observed reduction of mechanical hyperalgesia in CCI mice treated with BoNT/B, are not associated with an improvement in functional recovery, as evidenced by the similar weight bearing and similar sciatic static index observed in BoNT/B-treated CCI mice compared to untreated mice. Results from immunofluorescence experiments performed to measure the expression of proteins, such as SCs, peripheral myelin, mast cells, and macrophages, which are normally potentiated by BoNT/A during peripheral nerve regeneration, showed that the expression of these proteins was reduced or left unchanged by BoNT/B. In summary, together these data indicate that BoNT/B exerts analgesic effects on allodynia, similarly to BoNT/A, but does not exert beneficial action on functional recovery of the injured hind limb, reproducing only some of the effects induced by BoNT/A on structural alteration injured nerves. The different effects of BoNT/A and BoNT/B on the regeneration of peripheral injured nerves may reside in the different target of enzymatic activity of BoNT/A (i.e., SNAP-25 protein) and of BoNT/B (i.e., vesicle-associated membrane protein (VAMP)-2 protein, another protein of SNARE complex) and on the different localization of these proteins inside the cells involved in the processes. Although further research will be needed to clarify and better understand the molecular mechanisms underlying the different effects of BoNT/A and BoNT/B, data of Finocchiaro et al. [92] clearly demonstrate the not complete interchangeability of the two botulinum serotypes. This is particularly relevant in view of a therapeutic approach aimed at neutralizing the neural dysfunction induced by peripheral nerve injury.

## 3. Central Nervous System

As already mentioned in Section 1, this review does not consider all the possible lesions of the CNS but only the traumatic lesions of the spinal cord. Spinal cord injury (SCI) is one of the most debilitating neurological conditions, which represents a dramatic health and social challenge that needs attention by the medical and scientific community. The main causes of SCI are traumas from the road, sports, weapons, and work accidents. The clinical outcomes of SCI depend on the severity and location of the lesion and may include partial or complete loss of motor and/or sensory functions and associated comorbidities, such as neuropathic pain. Complete SCI is the most disabling form of SCI, characterized by no motor or sensory function below the level of injury [93]. Complete SCI at the cervical region, with the most common level affected being C5, is associated with quadriplegia while complete SCI at thoracic and lumbar regions causes paraplegia. Regarding the clinical classification system of SCI, many different scoring systems by which the severity of SCI could be measured, compared, and correlated with the clinical outcomes, have been proposed in the past. Starting in the 1980s, the American Spinal Injury Association scoring system was proposed, and is currently the most widely accepted and used clinical scoring system for SCI [94].

### 3.1. Pathophysiology of Traumatic SCI

**Figure 2:**
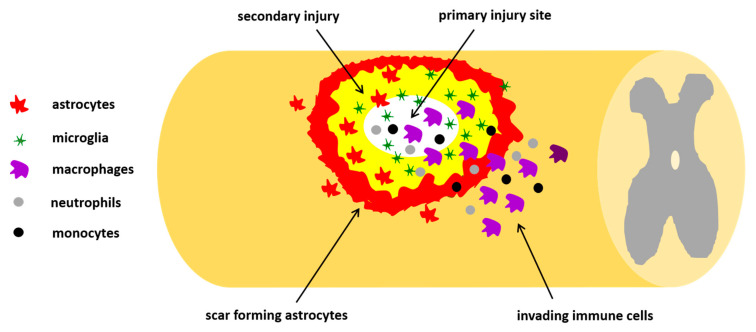
Schematic representation of traumatic spinal cord injury (SCI). The primary injury is the traumatic spinal lesion causing activation of many cellular and molecular mechanisms, including an inflammatory response, with invasion of neutrophils, monocytes, microglia, and macrophages, and a fibroblast response from different origins, forming the fibrotic scar. Altogether, the influx of these invading immune cells and macrophages leads to the formation of a fluid-filled cavity, which brings about the secondary injury, causing the upregulation of reactive astrocytes which form the astrocytes scar. The glial scar then acts as an inhibitory factor leading to myelin degradation and nerve conduction loss.

Pathophysiology of SCI is characterized by primary and secondary phases. The primary phase is related to the mechanical impact on the spine that fractures or dislocates vertebrae. Depending on the intensity of damage, primary injury can be divided as impact alone with transient compression, impact with persistent compression, distraction, and, finally, laceration/transection of spinal cord. The secondary phase is characterized by an acute, an intermediate, and a chronic phase. In the acute phase, starting a few minutes after trauma, pathophysiological changes (edema, thrombosis, inflammation, and neuronal excitotoxicity) occur, giving rise to neuroinflammatory response. In the intermediate stage, from days to weeks post-injury, cell degenerative mechanisms, including demyelination, apoptosis, glial and macrophage activation, gradually spread out from the lesion epicenter to uninvolved neural areas causing further loss of nervous tissue (Figure 2). All these phenomena create a loop: on one hand the clearance of tissue debris appears crucial to combat the damage; on the other hand, the excessive release of proinflammatory agents from activated and hyperreactive glia causes cell death. The formation of glial scar surrounding the necrotic part of the epicenter represents a physical barrier that hampers the neural function and axonal regeneration. The chronic phase is characterized by maturation of the lesion with glia scar and syrinx development [95].

### 3.2. Mechanisms of Regeneration after SCI

Various reasons determine the extreme limitation of spontaneous regeneration after SCI: (i) intrinsic factors, such as the absent reproductive capacity of adult neurons, due to the fact that they do not express a series of factors or molecular signals necessary to enter in a state of active growth; (ii) extrinsic factors, such as the unfavorable environment that is generated after an injury due to the wide range of inhibitory molecules that are secreted in extracellular matrix and in myelin sheaths; (iii) proliferation of glial cells, such as astrocytes and microglia, which produce proteins that prevent neuronal regeneration; and iv) secondary phenomena which develop as a consequence of the trauma [96]. The increase in factors such as the transforming growth factor (TGF), the fibroblast growth factor (FGF), interleukins, and the insulin-like growth factor (IGF-1) and thanks to the activation of the glial precursors, quiescent in physiological conditions, the microglial cells proliferate in a hypertrophic way; they give rise to a physical barrier, similar to the scar of fibroblasts that develops in peripheral tissues, called glial scar, which has the purpose of repairing damage and isolating any harmful material in contact with the damaged neurons. Together with the physical impediment that represents the glial scar in the development of axons, the astrocytes produce a series of inhibitory molecules, including the semaphorin and different ephrins, defining blocks beyond which axons cannot grow. Oligodendrocytes generate myelin, Nogo-A protein, and other membrane-associated glycoproteins (MAG) capable of blocking the growth of axons. The extracellular spaces of the scar also express many inhibitory factors, such as tenascin and proteoglycans chondroitin sulfate. In summary, the lack of regeneration after an injury in the adult central nervous system depends on many factors, the action of which results in a lack of induction of neuroplasticity, necessary to reshape axonal connections [96]. However, recent preclinical and clinical findings have shown that some degree of neurological recovery is possible [97], forcing a reconsideration of the principle of non-regeneration of the CNS after injury.

Many strategies for regeneration of adult CNS have been proposed, both from animal and human studies [96,98], including cell replacement by using stem cells [26,99], stimulation of self-repair by neural stem cells resident [100,101], neurotrophic factor delivery [102], axon guidance and removal of growth inhibition [103,104], manipulation of intracellular signaling, bridging and artificial substrates, and modulation of immune response [105]. Due to the complexity of the molecular and cellular environment of the SCI, an ideal drug should be able to simultaneously target different components to permit spontaneous neural circuits restoration. Under these premises, for its intrinsic properties, the botulinum neurotoxin seems to be a good candidate as a new therapeutic tool for counteracting SCI.

### 3.3. Botulinum Toxin and Spinal Cord Injuries

The botulinum toxin in a medical context relevant for therapeutic treatment of SCI has so far been considered for the treatment of urological complications related to spinal trauma at the lumbosacral level. Since bladder functions are regulated by lumbosacral innervations, injections of BoNT/A in the detrusor muscle has been proven effective in eliminating the obstructive mechanism, reducing neurogenic detrusor overactivity, increasing bladder capacity, and reducing urge incontinence, ensuring bladder emptying and urinary incontinence in patients with spinal cord injury [106,107]. 

Another established therapeutic use of botulinum toxin in SCI regards the pharmacological therapy of spasticity, a complication often associated with spinal trauma characterized by an abnormal muscle contraction of the limbs [108,109,110,111]. Injection of BoNT/A on specific muscles has been reported to improve mobility by counteracting spasticity in patients with spinal cord lesions [112,113,114,115,116]. Spasticity is often accompanied by a concomitant pain status in SCI patients, 60–70% of which experience nociceptive and neuropathic pain [117,118]. Although the correlation between spasticity, locomotor function, and pain is not linear and multiple factors may be involved, the injection of BoNT/A has become a first-line treatment for spasticity, and the improvement of spasticity may be often associated with an improvement of locomotion and pain [119,120].

Different from the many evidences about positive outcomes from use of BoNT/A for the treatment of spasticity associated to SCI (but not only), no evidences of BoNT/A effects directly on spinal regeneration after SCI have been reported till now. Recently, an experimental study analyzed the effect of BoNT/A on spinal regeneration and functional recovery after spinal traumas [121,122]. The authors considered two different mouse models of SCI: (i) a contusion model, of moderate trauma, with laminectomy [123,124] and (ii) a new validate mouse model of severe contusion without laminectomy [125]. The severe trauma induces a long-lasting paralysis and pain insensitivity and allows to evaluate the effects of BoNT/A on motor and sensitivity recovery, neuroprotection, and axonal regeneration; the moderate trauma, with laminectomy, induces a short-term paralysis and, in addition to severe trauma, allows to evaluate the neuropathic pain development as well. A single dose of BoNT/A (150 KDa purified protein without accessory proteins), at the same concentration as used in peripheral administration [57,59], was spinally administered within one hour from contusion. Toxin administration was performed between L4–L5 lumbar vertebrae and not at the injury site (T10–T11) to avoid systemic toxin diffusion due to hemorrhagic events following the contusion.

BoNT/A improves motor control, measured by Basso mouse scale [126] and restores thermal sensitivity, measured by tail-flick latency, both in severe and moderate mouse models of SCI. Following the severe trauma, a complete absence of hindlimb movement was observed after SCI, both in BoNT/A-treated and -untreated SCI mice. Very surprisingly, four days after SCI, a gradual motor improvement was observed in BoNT/A-treated mice followed by a complete recovery of normal motor performance and thermal sensitivity within 30 days while, as expected from severe trauma, untreated SCI mice remained paralyzed and thermally insensitive at all time points.

Neuropathic pain is a comorbidity frequently associated to SCI. To evaluate the BoNT/A effects in counteracting the onset of neuropathic pain, Marinelli et al. [121] used a moderate SCI trauma model in which nociceptive sensitivity was partly maintained in SCI mice differently from a severe SCI trauma model in which control mice never recovered sensitivity (see also [122]). After SCI, control untreated mice immediately developed allodynia which was maintained for the entire test period. BoNT/A treatment in SCI mice efficiently affected neuropathic pain onset.

All these behavioral outcomes from the BoNT/A treatment of SCI mice were accompanied by a series of cellular, tissue, and functional adaptation compared to untreated SCI mice. These adaptations included: (i) recovery of muscle atrophy as a consequence of motor neurons reconnection, as revealed by the increased staining with anti-bungarotoxin and neurofilament-L, as well as the anatomical integrity of nerve structures in skeletal muscle; (ii) modulation of glia scarring and reaction, as revealed by morphometric analysis of astrocytes, revealing hyperactive glial cells in perilesioned areas of saline mice, while only a slight reaction in BoNT/A mice, confirmed by dimensional analysis showing reduction in glial cell size in both perilesioned and epicenter areas of BoNT/A mice; (iii) protective effects of BoNT/A on lipid and glycemic profiles, with preservation of normoglycemic level, and on cell death and remyelination, with preservation of myelin basic protein (MBP) in BoNT/A mice, supporting a reduced reaction of oligodendrocytes, probably due to a minor degree of degeneration; (iv) pro-regenerative effects of BoNT/A in stem cells stimulation and 3D spinal cord reconstruction. In other words, BoNT/A treatment in SCI mice was able to reduce tissue damage and neuronal loss and to promote motoneuron survival; it was stimulating to determine its effects on stem cell proliferation. The possibility that a pharmacological treatment can stimulate endogenous stem cells in the adult spinal cord and promote functional recovery paves the way for developing new therapeutic strategies aimed at stimulating endogenous spinal cord stem cells for SCI repair.

Altogether these findings reveal the extraordinary ability of BoNT/A, never demonstrated before, in neuroprotection and promotion of spinal cord regeneration in a mouse model of SCI. Although the comprehension of all molecular events responsible for this regeneration ability needs to be deeply elucidated, and further investigations are requested, the study of Marinelli et al. [121] opens a new scenario in the therapy and care of spinal lesions and encourages clinical translation. Since pharmacology, safety, and toxicity of BoNT/A are well documented, these findings strongly encourage the clinical translation.

## 4. Conclusions

Although much more research is needed to better understand the phenomena associated with nerve regeneration, the experimental data presented in this review represent a stimulus to further continue in characterizing the potential use of botulinum toxin as a therapeutic agent capable of promoting the processes involved in nervous regeneration. Of particular medical importance is the possibility of use of botulinum toxin in neuronal regeneration after spinal cord injury, for which current therapeutic approaches are not sufficient to achieve complete restoration of health conditions before spinal trauma.

## 5. Patents

Patent n. US2019/0224288A1.
